# Is Off-Frequency Overshoot Caused by Adaptation of Suppression?

**DOI:** 10.1007/s10162-014-0498-0

**Published:** 2014-12-03

**Authors:** Mark Fletcher, Jessica de Boer, Katrin Krumbholz

**Affiliations:** Nottingham, UK

**Keywords:** enhancement, Zwicker tone, two-tone suppression, temporal effect, lateral inhibition, medial olivocochlear (MOC) system

## Abstract

This study is concerned with the mechanism of off-frequency overshoot. Overshoot refers to the phenomenon whereby a brief signal presented at the onset of a masker is easier to detect when the masker is preceded by a “precursor” sound (which is often the same as the masker). Overshoot is most prominent when the masker and precursor have a different frequency than the signal (henceforth referred to as “off-frequency overshoot”). It has been suggested that off-frequency overshoot is based on a similar mechanism as “enhancement,” which refers to the perceptual pop-out of a signal after presentation of a precursor that contains a spectral notch at the signal frequency; both have been proposed to be caused by a reduction in the suppressive masking of the signal as a result of the adaptive effect of the precursor (“adaptation of suppression”). In this study, we measured overshoot, suppression, and adaptation of suppression for a 4-kHz sinusoidal signal and a 4.75-kHz sinusoidal masker and precursor, using the same set of participants. We show that, while the precursor yielded strong overshoot and the masker produced strong suppression, the precursor did not appear to cause any reduction (adaptation) of suppression. Predictions based on an established model of the cochlear input–output function indicate that our failure to obtain any adaptation of suppression is unlikely to represent a false negative outcome. Our results indicate that off-frequency overshoot and enhancement are likely caused by different mechanisms. We argue that overshoot may be due to higher-order perceptual factors such as transient masking or attentional diversion, whereas enhancement may be based on mechanisms similar to those that generate the Zwicker tone.

## INTRODUCTION

The ability to hear out a signal from a background sound can be improved by a preceding sound (henceforth referred to as precursor). One instance of this kind of context-dependent change in signal audibility is the so-called “overshoot” or “temporal” effect. Overshoot refers to the fact that the detectability of a signal can be degraded when it is presented at the onset of a masker, rather than after a delay (Zwicker 1965a). Another instance of context-dependent change in signal audibility is “enhancement,” which refers to the phenomenon whereby a spectral region in a complex sound “pops out” (i.e., becomes more salient) when that region is preceded by its spectral complement (Schouten 1940; Viemeister 1980).

Overshoot is observed only when the signal is shorter than about 20 ms (Fastl 1977). In contrast, enhancement persists even when the signal is hundreds milliseconds long (e.g., Summerfield et al. 1984; Carlyon 1989; Thibodeau 1991). Measurements of enhancement have used precursors with energy both above and below the signal frequency, but no, or reduced, energy at the signal frequency (e.g., Viemeister et al., 2013). In contrast, overshoot is typically measured using broadband precursors and maskers, with energy both at and away from the signal frequency. However, it has been shown that substantial overshoot is also observed when the precursor and masker are narrowband, but only when their frequency is sufficiently different from the signal frequency (Zwicker 1965b; Bacon and Smith 1991). With broadband precursors and maskers, it is thought that both the on- and off-frequency energy within them contribute to overshoot (henceforth referred to as “on-” and “off-frequency overshoot”), albeit by different mechanisms. The mechanism proposed to underlie off-frequency overshoot (Strickland 2004, 2008) is similar to that proposed to underlie enhancement (Viemeister and Bacon 1982): In both phenomena, the signal response is thought to be increased in the presence of the precursor, because suppression of the signal by the masker is reduced, or “adapted.” In off-frequency overshoot, the suppression by the masker is thought to occur in the cochlea (referred to as two-tone suppression), and the adaptive effect of the precursor is thought to be mediated by the medial olivocochlear (MOC) system (Strickland, 2004). In enhancement, suppression and adaptation of suppression are thought to occur more centrally, possibly involving neural adaptation and lateral inhibition (Palmer et al. 1995; Wright 1996; Nelson and Young 2010). For enhancement, the adaptation-of-suppression hypothesis has been tested explicitly. In particular, it has been shown that an enhanced signal causes more forward masking than an unenhanced signal, indicating that it elicits a larger response (Viemeister and Bacon 1982; Thibodeau 1991; Byrne et al. 2011). For off-frequency overshoot, however, the adaptation-of-suppression hypothesis has not yet been explicitly tested.

The aim of the current study was to conduct this test. The most off-frequency overshoot is produced when the masker (and precursor) frequency is higher than the signal frequency (Schmidt and Zwicker 1991). Higher-frequency maskers also produce more suppression (referred to as high-side suppression) than lower-frequency maskers (low-side suppression; Shannon 1976; Duifhuis 1980; Cooper 1996). Psychophysical and physiological studies have shown that low-side suppression grows roughly linearly with masker level, whereas high-side suppression grows compressively (Duifhuis 1980; Javel et al. 1983; Costalupes et al. 1987; Delgutte 1990; Cooper 1996; Yasin and Plack 2007). This suggests that low-side suppression is caused by the tail and high-side suppression by the peak of the masker’s travelling wave response (see Patuzzi 1996, for a detailed discussion of this hypothesis). The peak amplitude of the travelling-wave response depends on the amount of cochlear amplification (Robles and Ruggero 2001). Thus, when the masker frequency is higher than the signal frequency, a reduction in the masker amplification through activation of the MOC system should reduce the amount of suppression caused by the masker, which would, in turn, increase the response to the signal.

**FIG. 1:**
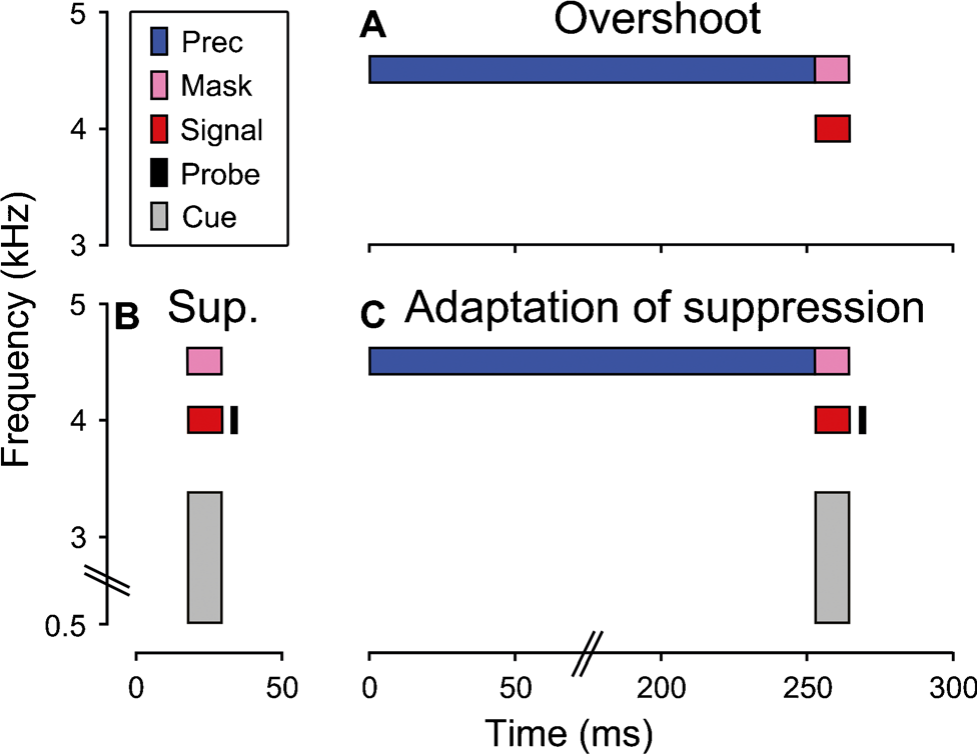
Schematic representation of the spectral and temporal characteristics of the stimuli used in the overshoot (**A**), suppression (**B**) and adaptation of suppression (**C**) experiments. The different stimuli (signal, probe, masker and precursor) are represented by different colors (see legend in panel **A**). The overshoot experiment used a 2.5- and 12.5-ms signal duration; only the 12.5-ms duration is shown here. The precursor duration is not to scale.

In the current study, we measured overshoot for a short sinusoidal signal at 4 kHz, with a sinusoidal masker and precursor at 4.75 kHz (Fig. 1a). To maximize the chances of finding overshoot in all individuals, the precursor and masker were presented continuously, without a gap. This means that at least some part of the measured overshoot may have been caused by a reduction in central masking effects, such as transient masking (Bacon and Moore 1987) or diversion of attention (Scharf et al. 2008) by the masker onset: Without the precursor, the masker onset could be confused with the signal onset or draw attention away from the signal frequency towards the masker frequency. Continuous presentation of the precursor and masker removes the masker onset and thus eliminates these effects, making the signal more clearly audible. In order to test whether any part of the measured overshoot was caused by adaptation of suppression, we measured the suppression of the signal by the masker both with and without the precursor present. For that, we first measured the forward-masking effectiveness of the signal alone and the signal and masker combined. A reduction in the forward-masking effectiveness of the signal by the masker would be assumed to be indicative of suppression (see, e.g., Houtgast 1972; Shannon 1976). Importantly, the signal and masker durations were chosen so that the signal and masker would not have been able to elicit the MOC system in time to influence the amount of forward masking (12.5 ms; see Wojtczak and Oxenham 2010). Then, we measured the forward-masking effectiveness of the signal when presented together with the masker and the precursor. A reduction in suppression due to the precursor (adaptation of suppression) would be expected to increase the forward-masking effectiveness of the signal.

In principle, our approach is similar to that used by Viemeister and Bacon (1982) for enhancement. There are, however, crucial differences. Firstly, their precursor and masker were broadband with a spectral notch, whereas ours were sinusoidal. Secondly, their signal and masker were almost ten times longer than ours (100 versus 12.5 ms). Other studies on enhancement have used even longer signals and maskers (Thibodeau 1991). Whereas Viemeister and Bacon found evidence for adaptation of suppression in enhancement, we found no such evidence in overshoot, despite finding substantial overshoot and suppression. Predictions based on an established model of the cochlear input–output (IO) function indicate that our failure to find adaptation of suppression in overshoot is unlikely to represent a false-negative outcome.

## METHODS

### General Outline

This study consisted of three experiments. In the first experiment, referred to as the “overshoot experiment” (Fig. 1a), overshoot was measured for a 4-kHz sinusoidal signal and a sinusoidal masker and precursor at one auditory-filter bandwidth [equivalent rectangular bandwidth (ERB); Glasberg and Moore 1990] above the signal frequency (4.75 kHz). The precursor and masker had durations of 252.5 and 12.5 ms, respectively, and were presented continuously and at the same level. The signal had a duration of 2.5 ms, initially, and was gated on together with the masker. The 2.5-ms duration was used, because shorter signal durations have yielded larger overshoot effects in previous studies (Zwicker 1965b). Subsequently, we remeasured overshoot with a 12.5-ms signal duration (referred to as “supplementary overshoot experiment”), because this was the signal duration in the other two experiments. In this case, the signal and masker were gated on and off together. In both overshoot experiments, the signal detection threshold was measured both with and without the precursor present. Overshoot is the difference in signal detection threshold between these two conditions.

Given that, in the overshoot experiment, the masker frequency was higher than the signal frequency, the masking effect would be presumed to have been predominantly suppressive (e.g., Duifhuis 1980; Cooper 1996). The second experiment, referred to as the “suppression experiment,” used a forward-masking paradigm to quantify the amount of suppression exerted by the masker (Fig. 1b). In the suppression experiment, the signal duration was 12.5 ms, and the detection threshold was measured for a sinusoidal probe stimulus, presented 2.5 ms after the signal offset. The probe had the same frequency as the signal and a duration of 2.5 ms. The probe detection threshold was measured in the presence of either the signal alone or the signal and masker combined. The signal and masker were gated on and off together. Any suppression exerted by the masker would decrease the size of the signal response, which, in turn, would decrease the probe detection threshold (i.e., make the probe easier to detect). The signal and masker levels were set individually for each participant. First, the signal detection threshold was measured in quiet, and the signal level was set to 25 dB sensation level (SL). Then, the masker level was set so that the masker would just render the 25-dB SL signal inaudible. In the overshoot experiment, the masker and precursor were set to the same level as in the suppression experiment. Stimuli of similar durations and levels as in the suppression experiment have been used previously to measure cochlear compression using forward-masking (Yasin et al. 2013).

According to the adaptation-of-suppression hypothesis, the precursor would be expected to reduce the amount of suppression exerted by the masker in the overshoot experiment. The aim of the third experiment, referred to as the “adaptation-of-suppression experiment,” was to quantify any precursor-induced reduction in suppression using the same forward-masking paradigm as used in the suppression experiment. Any precursor-induced reduction in suppression would increase the signal response and thus manifest as an increase in the probe detection threshold.

Control measurements were conducted to measure the probe detection threshold in the presence of the masker alone and the masker and precursor combined. This was to assess the direct masking effects of the masker and/or precursor on the probe. The timing of the stimuli was the same as in the suppression and adaptation-of-suppression experiments.

### Stimuli

All stimuli were gated on and off with 2.5-ms quarter-sine and quarter-cosine ramps, respectively $\left[ \sin \left(\frac{\pi }{2}\cdot \frac{t}{2.5}\right)\right.$ and $\cos \left(\frac{\pi }{2}\cdot \frac{t}{2.5}\right)$, where *t* is time in milliseconds]. All stated durations of stimuli and gaps between stimuli refer to the time between the 3 dB-down (half-power) points of the stimulus ramps. The phases of the masker, signal, and probe were randomized between trials. The phase of the precursor was set such that there was no phase discontinuity between the precursor offset and the masker onset.

In the suppression and adaptation-of-suppression experiments, a cue was presented to disambiguate the signal from the probe. The cue was gated on and off simultaneously with the signal. It was a 15-ERB wide noise, centered 9 ERBs below the signal frequency. It was filtered so as to produce equal excitation per ERB within its passband (Glasberg and Moore 2000) and presented at 30 dB SPL/ERB. It was confirmed that, at this level, the cue did not produce any significant masking of the probe.

When measuring the masker level needed to render the signal inaudible, it was difficult to hear out the signal when the masker and signal were gated on and off simultaneously. Therefore, in these measurements, the masker duration was increased so that the masker onset preceded the signal onset by 10 ms. The offsets remained simultaneous.

All stimuli were generated digitally at a sampling rate of 25 kHz using TDT System 3 (Tucker-Davies Technologies, Alachua, FL, USA) and MATLAB (The MathWorks, Natick, MA, USA). They were digital-to-analogue converted with a 24-bit amplitude resolution (TDT RP2), amplified (TDT HB7), and presented monaurally to the left ear using Sennheiser HD 600 headphones (Wedemark-Wennebostel, Germany). Participants were seated in a double-walled, sound-attenuating booth (IAC, Winchester, UK).

### Experimental Protocol

The study was conducted in four consecutive stages. Firstly, participants were screened for normality of hearing. Secondly, detection thresholds in quiet were measured for the probe and 12.5-ms signal used in the suppression and adaptation-of-suppression experiments. For the probe, the detection threshold was also measured in the presence of the cue stimulus (see “Stimuli”). Thirdly, the masker level needed to just render inaudible the 12.5-ms signal at 25 dB SL was measured. Finally, the conditions from the overshoot, suppression, and adaptation-of-suppression experiments, as well as the control experiment were measured in a random order. The supplementary overshoot experiment was measured after the other conditions, with a partially different set of participants.

Overall, the study lasted around 10 h, depending on the amount of training needed for performance to stabilize. The study was conducted over several days and included regular breaks.

### Procedure

All thresholds were measured using a three-interval, three-alternative forced-choice adaptive tracking procedure. One of the three intervals, the target interval, contained the stimulus that was to be detected (i.e., the signal in the overshoot experiment and the probe in the suppression and adaptation-of-suppression experiments) with equal a priori probability. The task was to select the target interval by pressing the appropriate response button. The intervals were 272.5-ms long, cued visually, and separated by 500-ms gaps. The adaptive parameter was the signal level in the overshoot experiment and the probe level in the suppression and adaptation-of-suppression experiments. It was varied adaptively according to a two-down, one-up rule, which tracks 70.7 % correct performance (Levitt 1971). The steps were 10 dB up to the first reversal, 5 dB up to the second reversal, and 2.5 dB for the remaining eight reversals that made up each track. Visual feedback was given after each trial. The last six reversals in each track were averaged to estimate threshold. For each condition and participant, tracks were run until the average of the last three threshold estimates had a standard error of less than 1.5 dB. The average of the last three threshold estimates was taken as the overall estimate.

In the measurements for setting the masker level, the stimulus to be detected was the signal, and the adaptive parameter was the masker level. The masker level was varied according to a two-up, one-down tracking rule. In all other respects, the procedure was the same as stated above.

### Participants

A total of seven participants (four male, aged between 20–28 years) were tested. They were screened for normal hearing (absolute thresholds ≤20 dB HL), had no reported history of audiological or neurological disease, and were not taking any neuroactive medication. Five participants took part in the original overshoot experiment, with the 2.5-ms signal duration, as well as in the suppression and adaptation-of-suppression experiments. Four participants (two new) took part in the supplementary overshoot experiment, with the 12.5-ms signal duration. One participant took part in the piloting for this study, the others had no previous psychoacoustic experience.

Written informed consent was obtained from all participants. The experimental procedures were approved by the Ethics Committee of the Nottingham University School of Psychology and conformed to the guidelines of the Declaration of Helsinki at the time the data were collected (version 6, 2008) but was not formally pre-registered online in accordance with the 2014 amendment to the Declaration. Participants were paid at an hourly rate.

## RESULTS

### Overshoot Experiment

**FIG. 2:**
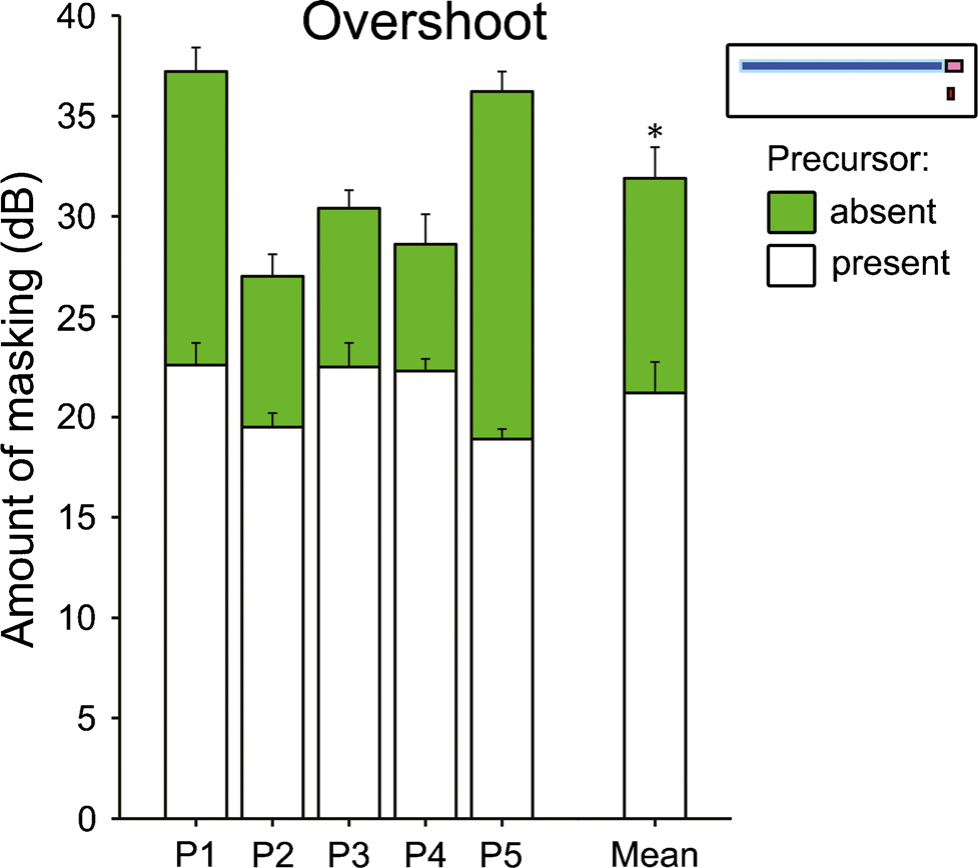
Individual (*left bars*) and average (*right bars*) signal detection thresholds from the overshoot experiment. The *green bars* in the background show the thresholds when the precursor was absent and the *white bars* in the foreground show the thresholds when the precursor was present. The overshoot is indicated by the visible portion of the *green bars*. All thresholds are expressed as amount of masking, that is, the masked threshold relative to the threshold in quiet. The *error bars* show the standard errors (SE). For the average, the SE was corrected for across-participant variability using the method proposed by Morey (2008). The stimulus configuration is shown in the inset (top right-hand corner).

In the overshoot experiment, all five participants showed substantially lower masked signal detection thresholds with than without the precursor present, indicating overshoot (Fig. 2). The average overshoot amounted to 10.7 ± 2.2 dB (mean ± standard error) and was statistically significant [paired *t* test (two-tailed), *t* (4) = 4.9, *p* = 0.008]. The variation in the amount of overshoot across participants was considerable but consistent with previous studies (e.g., Strickland 2004). The masker level was set individually for each participant, so that it would just render the 25-dB SL signal used in the suppression and adaptation-of-suppression experiments inaudible (see Table 1). On average, the masker level was 76.6 ± 1.2 dB SPL. The precursor level was the same as the masker level (see “METHODS”).

**TABLE 1:** Quiet detection threshold of the 2.5-ms probe (left column) and sound pressure level (SPL) of the 12.5-ms signal at 25 dB SL (middle column). The probe and 25-dB SL signal were used in thesuppression and adaptation-of-suppression experiments. The right column shows the masker levelneeded to just render the 25-dB SL signal inaudible (used in all experiments). Individual and averagevalues with standard errors (SEs) are shown in different rows

Participant	2.5-ms probe quiet threshold (dB SPL)	12.5-ms signal SPL level at 25 dB SL	Masker level (dB SPL)
P1	26	45	77
P2	33	53	75
P3	27	47	80
P4	32	53	78
P5	21	41	73
Mean ± SE	27.8 ± 2.2	47.8 ± 2.3	76.6 ± 1.2

### Suppression Experiment

**FIG. 3:**
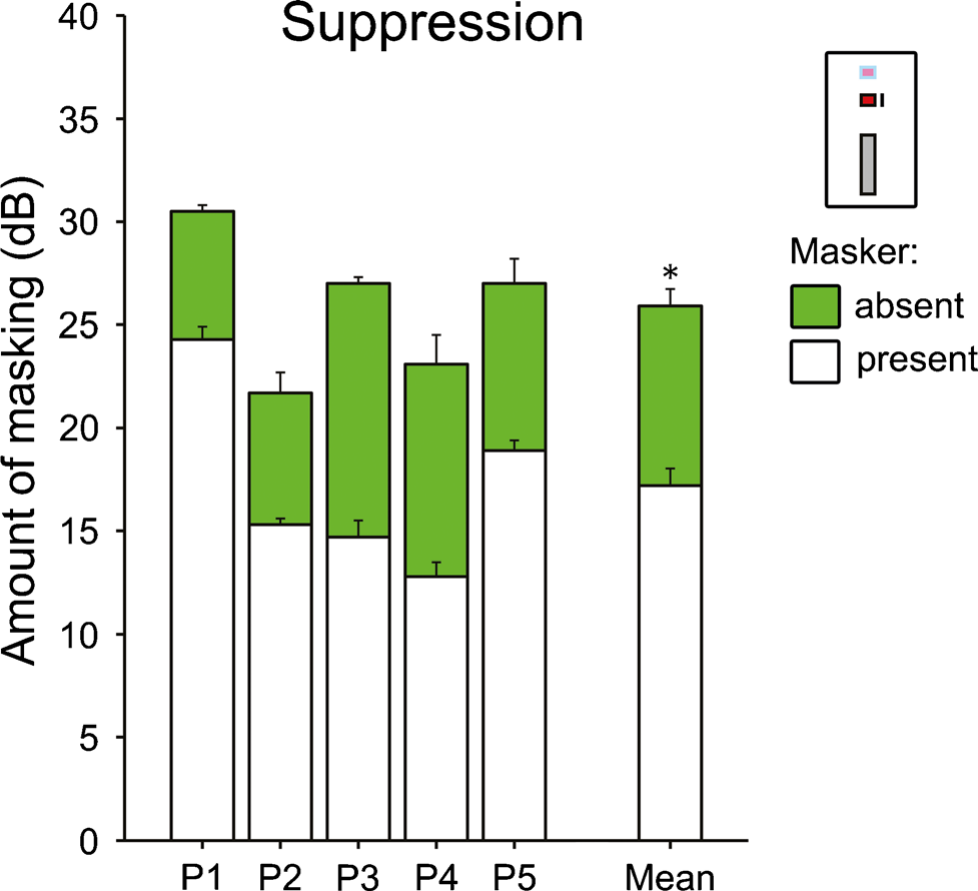
Individual (*left bars*) and average (*right bars*) probe detection thresholds from the suppression experiment. The *green bars* in the background show the thresholds when the probe was masked by the signal alone and the *white bars* in the foreground show the thresholds when it was masked by the signal and masker combined (see inset in right-hand corner). The visible portion of the *green bars* shows the suppression of the signal by the masker. As in Fig. 2, all thresholds are expressed as amount of masking and the *error bars* show the SE (corrected for across-participant variability for the average).

Given that substantial overshoot was found in the overshoot experiment and that, according to the adaptation-of-suppression model, overshoot is due to suppression being stronger when the precursor is absent than when it is present, it would be expected that, in the absence of the precursor, there would be substantial suppression. The suppression experiment showed that this was indeed the case. In all five participants, the signal caused considerably more forward-masking when it was presented alone than when it was presented together with the masker (Fig. 3). This suggests that the signal was being suppressed by the masker. On average, the probe detection threshold changed by 8.7 ± 1.2 dB; this was statistically significant [*t* (4) = 7.4, *p* = 0.002].

The amount of residual masking caused by the signal, when it was presented together with the masker and was thus inaudible, reveals whether the masker masked the signal exclusively by suppressive masking, or also by excitatory masking (Moore and Vickers 1997; Plack et al. 2006). If the masker effect were exclusively suppressive, the signal response would be at the quiet threshold, and so, there should be little or no residual masking by the signal. If, on the other hand, the masker effect were exclusively excitatory, the signal should cause as much residual masking as when presented alone. The current data showed no significant residual masking [defined as the difference between the probe detection thresholds for the signal and masker combined and the masker alone, which was 2.1 ± 1.3 dB on average; *t* (4) = 1.7, *p* = 0.172]. This suggests that the masker effect on the signal was predominantly suppressive. Despite causing mainly suppressive masking of the simultaneous signal, the masker alone caused considerable forward-masking of the subsequent probe [15.5 ± 3.2 dB on average].

The suppression experiment involved a cue stimulus to disambiguate the signal from the probe. A control measurement showed that the cue itself did not cause any significant masking of the probe [average amount of masking by the cue = 0.9 ± 0.7 dB, which was not statistically significant; *t* (4) = −0.9, *p* = 0.204].

The difference between the probe detection thresholds for the signal alone and for the signal and masker combined might be much larger than the actual suppression in cochlear gain exerted by the masker. This is because the cochlear IO function of the probe is compressive for mid-range levels, and so, a small change in gain might yield a larger change in probe detection threshold. Here, we use an established model of the cochlear IO function to convert the change in probe detection threshold into an estimate of the actual change in cochlear gain. The model assumes that the cochlea applies active amplification at, and within a narrow range around, the characteristic frequency, but not at more remote frequencies (Rhode 1971). It is assumed that the amplification is maximal at low sound levels, decreases progressively at medium sound levels, and is absent at high sound levels. The amplification is assumed to apply instantaneously. This general model has been successfully used to fit psychophysical estimates of the auditory filter widths as a function of sound level (Glasberg and Moore 2000) and to derive psychophysical estimates of cochlear compression using various types of experimental paradigms (e.g., Plack and Arifianto 2010; Yasin et al. 2013).

**FIG. 4:**
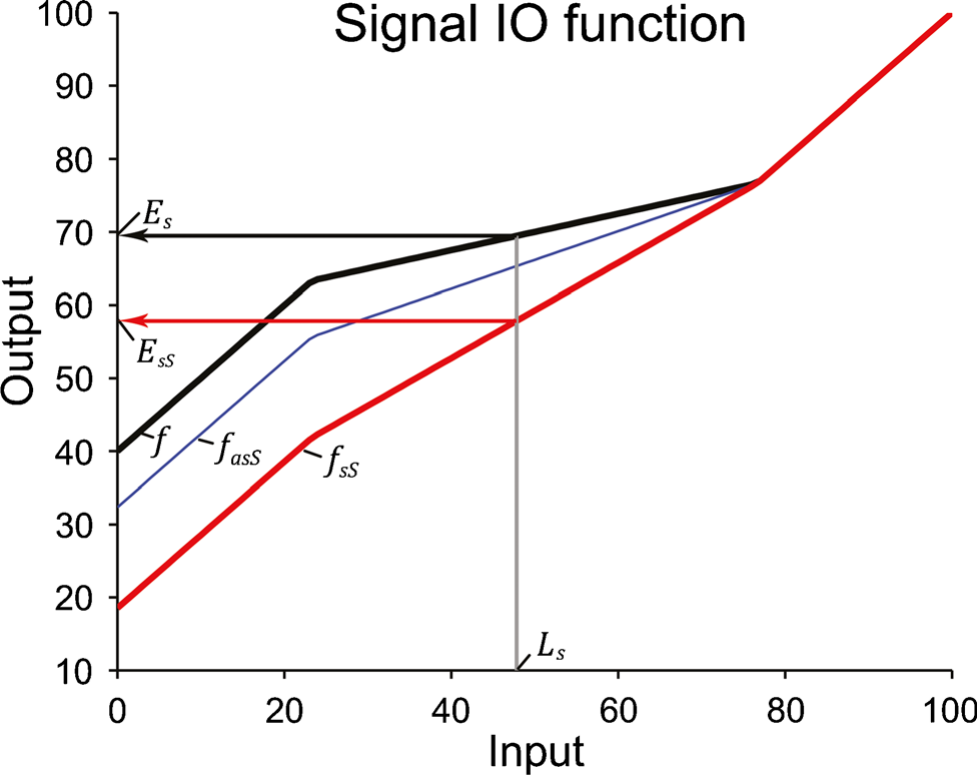
Simulated cochlear input–output (IO) functions of the signal in the suppression experiment. The *bold black line*, labelled *f*, shows the IO function when the signal is presented alone and is thus unsuppressed. The *bold red line*, labelled *fsS*, shows the IO function when the signal is presented together with the masker and is thus suppressed. The *grey vertical line* shows the average sound pressure level of the signal. The horizontal *black and red arrows* show the response levels of the unsuppressed (*E*_*S*_) and suppressed signal (*E*_*sS*_), respectively. The *dashed blue line*, labelled *f*_*asS*_, shows the simulated signal IO function in the presence of both the masker and precursor (to be discussed in the adaptation-of-suppression experiment). In this example, it was assumed that all of the measured overshoot was caused by adaptation of suppression. The model results shown here are based on the averaged data across participants and are for illustration only. The model predictions presented in the text are based on the individual data.

In the current implementation of this model, which is similar to the one used by (Yasin and Plack 2003), the cochlear IO function, *f*, was expressed as the sum of the sound level, *L*, and a level-dependent gain, *G*(*L*); in units of intensity: *f*(*L*) = 10^(*L* + *G*(*L*))/10^ (Fig. 4, black line). At low sound levels up to a first break point, *BP*_1_, the gain was assumed to be constant at the maxim value *G*_max_:

1 $$ G\left(L\le B{P}_1\right)={G}_{\max } $$

Between, *BP*_1_ and a second break point, *BP*_2_, the gain was assumed to decrease linearly from *G*_max_ to zero, at a rate of 1–*c*, where *c* is the compression exponent:

2 $$ G\left(B{P}_1\le L\le B{P}_2\right)=\left(c-1\right)\left(L-B{P}_1\right)+{G}_{\max } $$

The compressive range was assumed to be symmetric about *L* = 50 dB SPL, so *BP*_1_ = 50 − *G*_max_/2(1 − *c*) and *BP*_2_ = 50 + *G*_max_/2(1 − *c*). Above *BP*_2_, the gain was assumed to be zero:

3 $$ G\left(L\ge B{P}_2\right)=0 $$

Based on psychophysical data from humans (Nelson and Young 2010; Yasin et al. 2013) and physiological data from chinchillas (Ruggero et al. 1997), *G*_max_ was set to 40 dB, and *c* was set to 0.25. This meant that *BP*_1_ and *BP*_2_ were equal to 23.3 and 76.7 dB SPL, respectively. *f* was used to calculate the masking effect, *W*, of the signal alone (*WS*), the masker alone (*W*_*M*_) and the signal and masker combined (*W*_*SM*_); the masking effect corresponds to the cochlear response to the probe at the respective probe detection threshold, *L*_Pr *obe*_ : *W* = *f*(*L*_Pr *obe*_). The residual masking effect of the suppressed signal was calculated as the difference between the masking effects of the masker alone and the masker and signal combined: *W*_*sS*_ = *W*_*SM*_ − *W*_*M*_. *W*_*sS*_ was used to calculate the cochlear response to the suppressed signal, *E*_*sS*_, by assuming that *E*_*sS*_ and *W*_*sS*_ are related through a constant factor, *k*, which represents the signal-to-noise ratio at masked threshold: *E*_*sS*_ = *W*_*sS*_/*k. k* was derived from the masking effect of the signal alone, *W*_*S*_, and the cochlear response to the signal alone, *E*_*S*_ : *k* = *W*_*S*_/*E*_S_. *E*_*S*_ was calculated by passing the signal level, *L*_*S*_ (47.8 ± 2.3 dB SPL; see Table 1), through the cochlear IO function, *f. f* was equal to 1.48 ± 0.15, on average. The suppressed signal excitation, *E*_*sS*_, was used to calculate the cochlear gain of the suppressed signal, *G*_*sS*_ by subtracting the signal level, *L*_*S*_ : *G*_*sS*_ = 10 log_10_(*E*_*sS*_) − *L*_*S*_. The suppression is equal to the gain of the signal alone, *G*(*L*_*S*_), minus the gain of the suppressed signal, *G*_*sS*_. The observed suppression depends, not just on the effectiveness of the suppressor (i.e., the masker in this case), but also on the level of the suppressee (i.e., the signal); the higher the suppressee’s level, the lesser its cochlear gain, and so, the lesser the suppression. A better measure of the suppressor’s effectiveness is thus its effect on the maximum cochlear gain, *G*_max_. In order to calculate the suppression in *G*_max_, we calculated the IO function of the suppressed signal, *f*_*sS*_ (Fig. 4, red line). For that, we minimized the squared difference between *f*_*sS*_(*L*_*s*_) and the response to the suppressed signal, *E*_*sS*_, by varying *G*_max_ using *lsqnonlin* in Matlab. The breakpoints were kept constant, and so, varying *G*_max_ also varied the compression exponent, *c*. The model was applied to the data from each participant separately.

According to this model, the masker suppressed the cochlear gain of the signal by, on average, 12.7 ± 2.8 dB. Figure 4 shows the cochlear IO function of the suppressed signal, *f*_*sS*_ (red line). The maximum gain of this IO function was equal to 16.6 ± 4.3 dB, on average, suggesting that the masker suppressed the maximum cochlear gain by 23.3 dB, or 58 %. As a result, the compression exponent increased from 0.25 to 0.69 ± 0.08.

In order to test how sensitive the estimated suppression was to the parameters of the cochlear IO function, *f*, we re-ran the model with a range of values for the maximum cochlear gain, *G*_max_, and compression exponent, *c. G*_max_ and *c* were varied orthogonally, with *G*_max_ ranging from 30 to 50 dB in 5-dB steps, and *c* ranging from 0.15 to 0.35 in steps of 0.05. Over these ranges, the estimated average suppression in the signal’s cochlear gain ranged between 11.32 and 14.73 dB. It has been suggested that a change in the maximum cochlear gain, *G*_max_ does not change the maximum compression, *c*, but instead, changes the lower bound of the compressive range, *BP*_*1*_ (Plack et al. 2004). Using this assumption instead of our assumption that the breakpoints remain fixed did not affect the estimated suppression.

### Adaptation-of-Suppression Experiment

**FIG. 5:**
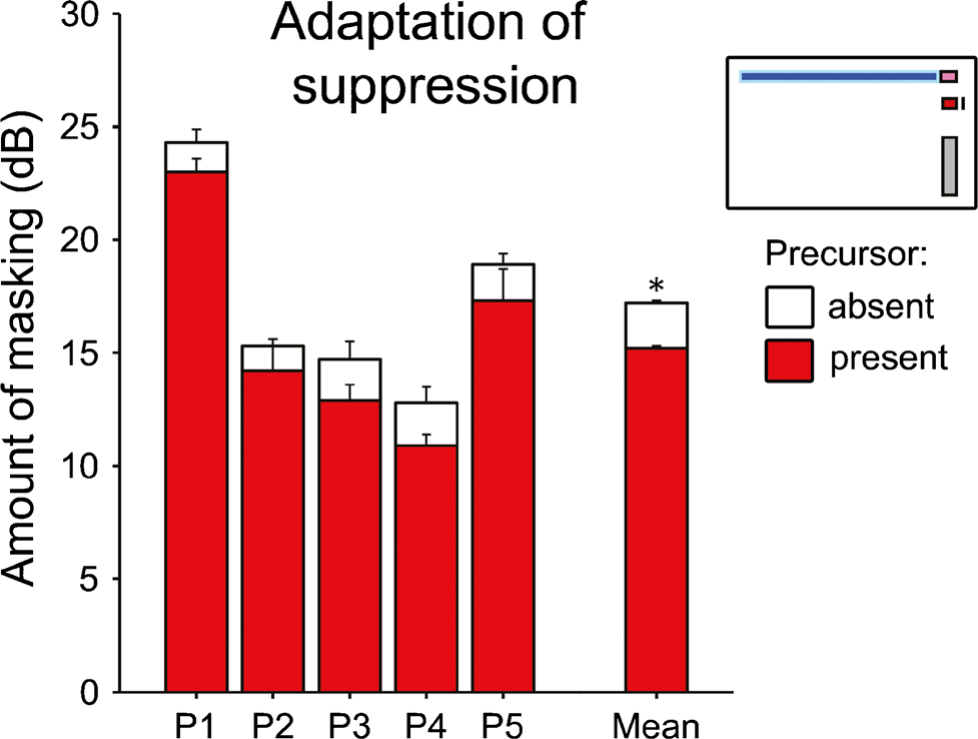
Individual (*left bars*) and average (*right bars*) probe detection thresholds from the adaptation-of-suppression experiment. The *white bars* in the background show the thresholds when the precursor was absent, and the *red bars* in the foreground show the thresholds when it was present (see *inset in right-hand corner*). As in Fig. 2, all thresholds are expressed as amount of masking, and the *error bars* show the SE (corrected for across-participant variability for the average).

According to the adaptation-of-suppression model of overshoot, the precursor should have reduced the suppressive masking of the signal by the masker. This should have increased the response to the signal and thus its forward-masking effect on the probe, causing an increase in probe detection threshold. This, however, was not observed. Instead of an increase, the precursor caused a small (1.5 ± 0.2 dB on average) but significant [*t* (4) = −10.2, *p* = 0.001] decrease in probe detection threshold (Fig. 5). A similar (0.9 ± 0.9 dB on average) albeit non-significant [*t* (4) = −1.0, *p* = 0.359] decrease in the probe detection threshold due to the precursor was also observed in the control experiment, where the probe detection threshold was measured in the presence of the masker alone, or the masker and precursor combined. The precursor effects in the adaptation-of-suppression and control experiments were not significantly different from one another [*t* (4) = −0.6, *p* = 0.573].

Does the failure to obtain an increase in probe detection threshold in the adaptation-of-suppression experiment represent a false-negative outcome? A false-negative outcome might have arisen if any precursor-induced increase in the cochlear gain of the signal (adaptation of suppression) produced a greater overshoot than change in probe detection threshold. In order to test this possibility, we used the same model of the cochlear IO function as in the suppression experiment to estimate the amount of change in probe detection threshold that would be expected for a given amount of overshoot caused by adaptation of suppression, *O*_*as*_. Any overshoot caused by adaptation of suppression would be associated with an increase in the cochlear gain of the signal. The remaining overshoot, *O*_*c*_, would be assumed to be due to release from central masking effects, such as transient masking or attentional diversion, and thus not be associated with any change in cochlear gain. The control experiment showed that the masker and precursor combined caused the same amount of forward-masking as the masker alone, indicating that the precursor caused no more excitatory masking than the masker alone. This suggests that the measured overshoot, *O*_meas_ reflects all of the overshoot that actually occurred, rather than some of the overshoot being counteracted by additional excitatory masking by the precursor. Thus,

4 $$ {O}_{meas}={L}_S(M)-{L}_S(MP)={O}_{as}+{O}_c $$

**FIG. 6:**
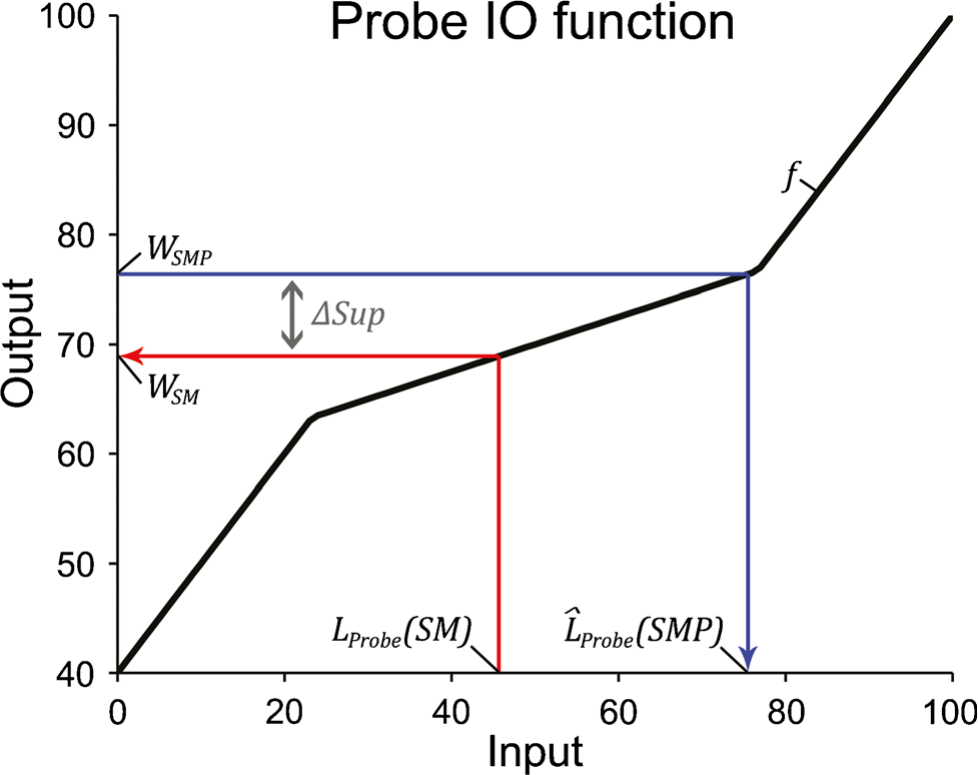
Simulated cochlear IO function, *f*, of the probe (*bold black line*) in the suppression and adaptation-of-suppression experiments. The *red arrow* connects the measured detection threshold, *L*_*Probe*_(*SM*) (input), and the simulated response level, *W*_*SM*_ (output), of the probe when it is masked by the signal and masker combined. The *blue arrow* connects the simulated response level, *W*_*SMP*_, and predicted detection threshold, ${\widehat{L}}_{\Pr obe}(SMP)$, of the probe when it is masked by the signal, masker, and precursor. In this example, it was assumed that all of the measured overshoot was caused by adaptation of suppression.

Here, *L*_*S*_(*M*) and *L*_*S*_(*MP*) are the signal detection thresholds for the masker alone and the masker and precursor combined. *O*_*as*_ was varied from zero to *O*_*meas*_, in 1-dB steps. First, we calculated the increase in the maximum cochlear gain of the signal, *G*_max_, associated with a given adaptation of suppression-related overshoot, *O*_*as*_, by calculating the cochlear IO function of the signal after adaptation of suppression, *f*_*asS*_. For that, we minimized the squared difference between the signal response with both central and adaptation of suppression-related overshoot taken into account, *f*_*asS*_(*L*_*S*_(*M*) − *O*_*as*_ − *O*_*c*_) = *f*_*asS*_(*L*_*S*_(*MP*)) [see Eq. 4] and the signal response with only central overshoot taken into account, *f*_*sS*_(*L*_*S*_(*M*) − *O*_*c*_). Here, *f*_*sS*_ is the cochlear IO function of the signal when it is fully suppressed by the masker, which was derived in the suppression experiment. In equating *f*_*asS*_(*L*_*S*_(*MP*)) and *f*_*sS*_(*L*_*S*_(*M*) − *O*_*c*_), we assumed that any increase in cochlear gain caused by adaptation of suppression would counteract the corresponding decrease in signal detection threshold, *O*_*as*_, to create a constant signal response at threshold. As in the suppression experiment, we varied *G*_max_ using *lsqnonlin* in Matlab, whilst keeping the breakpoints, *BP*_1_ and *BP*_2_, fixed [see Eqs. 1–3]. The dashed blue line in Fig. 4 shows *f*_*asS*_ if *all* of the measured overshoot had been caused by adaptation of suppression. We then calculated the actual gain of the signal in the adaptation-of-suppression experiment when both the masker and precursor were present, *G*_*asS*_. *G*_*asS*_ was calculated by substituting the signal level used in the adaptation-of-suppression experiment, *L*_*S*_, for *L*, and the maximum cochlear gain after adaptation of suppression, *G*_max_(*asS*), for *G*_max_ in Eqs. 1–3. *G*_*asS*_ was used to estimate the masking effect of the signal with both the masker and precursor present, *W*_*SMP*_. *W*_*SMP*_ was assumed to be equal to the masking effect of the signal with only the masker present, *W*_*SM*_, times a factor representing the increase in the signal gain as a result of adaptation of suppression, *ΔSup* = *G*_*asS*_ − *G*_*sS*_ : *W*_*SMP*_ = *W*_*SM*_ ⋅ 10^*ΔSup*/10^ (Fig. 6). The expected probe detection threshold with the signal, masker, and precursor present, ${\widehat{L}}_{\Pr obe}(SMP)$, was then estimated by passing *W*_*SMP*_ through the inverse of the cochlear IO function, *f* (Fig. 6, blue arrow). As in the suppression experiment, the model was applied to each participant’s data separately, and the results were averaged.

According to this model, every 1-dB increase in overshoot caused by adaptation of suppression would have been expected to be associated with an increase in probe detection threshold of, on average, 2.57 ± 0.15 dB in the adaptation-of-suppression experiment.

As in the suppression experiment, we re-ran the model with a range of values for the maximum cochlear gain, *G*_max_, and compression exponent, *c*. Again, *G*_max_ was varied between 30 and 50 dB in 5-dB steps, and *c* was varied between 0.15 and 0.35 in steps of 0.05. Over these ranges, the rate of increase in probe detection threshold with every 1-dB increase in adaptation of suppression-related overshoot ranged between 1.72 and 3.50 dB, on average. Using the assumption that the lower breakpoint, *BP*_1_, changes when *G*_max_ changes, rather than the compression exponent, *c*, increased the rate of increase in probe detection threshold per 1-dB increase in adaptation of suppression-related overshoot from 2.57 dB to 3.31 (±0.42) dB.

### Supplementary Overshoot Experiment

The signal was longer in the adaptation-of-suppression experiment (12.5 ms) than in the original overshoot experiment (2.5 ms). It is possible that the longer signal was less affected by the precursor than the shorter one, which would explain why we found overshoot but no adaptation of suppression. In order to test this possibility, we conducted a “supplementary overshoot experiment,” which measured overshoot for both the 2.5- and 12.5-ms signals. The procedures were the same as in the original overshoot experiment. Four participants (two from the original group and two new) took part in this experiment.

On average, we found 7.2 ± 1.9 dB of overshoot for the 2.5-ms signal, compared with 5.7 ± 0.8 dB for the 12.5-ms signal. This difference was not significant [paired *t* test: *t* (3) = 0.9, *p* = 0.410]. The overshoot for the 2.5-ms signal was smaller than that measured in the original overshoot experiment (10.7 ± 2.2 dB), albeit non-significantly [unpaired *t* test, *t* (7) = 2.0, *p* = 0.091]. Given that the stimuli were identical, this difference would appear to be due to variability between participants. The fact that the 2.5- and 12.5-ms signals yielded similar overshoot rules out the possibility that the failure to obtain adaptation of suppression was due to the difference in signal duration between the original overshoot and adaptation-of-suppression experiments.

## DISCUSSION

The adaptation-of-suppression model of overshoot posits that off-frequency overshoot arises because the precursor reduces suppressive masking of the signal by the masker. The reduction in suppression is thought to arise as a result of a reduction in the cochlear amplification of the masker (adaptation of suppression), which is thought to be mediated by the MOC system. In order to test this model, we measured overshoot, suppression, and adaptation of suppression using similar stimuli in the same set of participants. We found substantial overshoot and suppression in all participants, with effect sizes similar to those found in previous studies (e.g., Bacon and Moore 1986; Lee and Bacon 1998). Despite this, we found no evidence of adaptation of suppression, that is, no precursor-induced increase in probe detection threshold in the adaptation-of-suppression experiment. This suggests that adaptation of suppression did not appreciably contribute to the observed overshoot effect. Predictions based on an established model of the cochlear IO function showed that, due to the nonlinearity of the cochlear IO functions of the signal and probe, every decibel of overshoot caused by adaptation of suppression would have been associated with a precursor-induced increase in probe detection threshold of around 2.5 dB in the adaptation-of-suppression experiment. Thus, even if only 1 dB of the observed overshoot had been caused by adaptation of suppression, the precursor should have caused a detectable increase in probe detection threshold. Instead, it caused a small but significant decrease in probe detection threshold. A similar precursor effect was found in the control experiment, where the signal was not present. This suggests that the precursor effect in the adaptation-of-suppression experiment was caused by a central mechanism. The results of Scharf et al. (2008) suggest that the masker onset would have diverted attention away from the probe frequency towards the masker frequency and that the precursor would have mitigated this effect by giving the listener time to refocus attention back to the probe frequency.

There are three possible reasons as to why the precursor did not cause any measurable adaptation of suppression. Firstly, the precursor may not have caused any reduction in the cochlear amplification of the masker, either because it was spectrally too narrow to elicit the MOC system (Lilaonitkul and Guinan 2009a, b), or because the MOC effect occurred at a frequency other than the precursor (and thus masker) frequency (Lilaonitkul and Guinan 2012). Secondly, the precursor may have activated the MOC fibers at the masker frequency, but not much reduced the masker gain. This may have occurred because the masker was presented at a relatively high level (76.6 dB SPL on average), and so, the masker gain may have been low (Ruggero et al. 1997). Finally, the precursor may have elicited the MOC and caused a reduction in the masker gain, but this may not have reduced the suppression exerted by the masker.

The latter scenario would be predicted by the dual-filter model of suppression proposed by Plack et al. (2002). The dual-filter model of cochlear frequency selectivity describes the response of each point along the cochlear partition as the combination of two (tip and tail) filters (Goldstein 1989; Meddis et al. 2001). The tip filter simulates the amplified peak, and the tail filter, the passive tail, of the cochlear response. The tail filter is broader than the tip filter and centered at a slightly lower frequency. In Plack et al.’s model, suppression occurs within the tip filter. Importantly, in this model, the suppression *precedes* the cochlear amplification. This means that the amount of suppression is determined by the sound level of the suppressor and thus not influenced by its cochlear gain. Plack et al.’s model assumes the existence of a third filter, which does not seem to have any intended physiological correlate. This third filter is similarly broad as the tail filter, but centered at a frequency slightly *above* the tip filter. It enables the model to reproduce the difference in suppression threshold between low- and high-side suppressors (e.g., Shannon 1976; Cooper 1996). However, the model fails to predict the difference in the growth rate of suppression with suppressor level between low- and high-side suppressors (e.g., Duifhuis 1980; Delgutte 1990). Another approach to implement suppression in a dual-filter model, proposed by Goldstein (1990), does predict the difference in the suppression growth rate between low- and high-side suppressors. This is because Goldstein’s model assumes that suppression *follows* cochlear amplification, rather than preceding it. In Goldstein’s model, the suppressor and suppressee are processed through the same filter and are thus subject to the same cochlear gain. This means that any reduction in cochlear gain by the MOC system would affect the suppressor and suppressee equally, and so, as in Plack et al.’s model, little or no net effect on the amount of suppression would be expected. Like Plack et al.’s model, Goldstein’s model has to include a component with no intended physiological correlate; in this case, an expansive nonlinearity in the tail filter. This is to counter the effect of a compressive nonlinearity, which is applied to the combined tip and tail filter responses in order to produce suppression. While Goldstein’s model reproduces the difference in the growth rate of suppression between low- and high-side suppressors, it fails to predict the finding, from both physiological and psychophysical studies, that maximum suppression occurs at a frequency above, rather than at, the frequency of the suppressee (Arthur et al. 1971; Shannon 1976; Duifhuis 1980; Cooper 1996). This finding has led to the hypothesis that the active process (i.e., the group of outer hair cells) that amplifies the cochlear response is located basal to the response peak (see Patuzzi 1996). The suppressor is assumed to “jam” the suppressee’s active process. The effectiveness of this jamming is assumed to be determined by the size of the suppressor response at the place where the suppressee’s active process is located. This physiological model of suppression also explains the differences in both suppression threshold and suppression growth rate between low- and high-side suppressors. Transmission-line models, which try to emulate the physiological properties of the cochlea, correctly capture all the salient properties of suppression without any further assumptions (Epp et al. 2010). In transmission-line models, suppression arises as a result of interactions between different frequency channels with independent active processes. As a result, transmission-line models would be expected to predict that reducing the gain of a high-side suppressor, for instance, through elicitation of the MOC, would reduce the amount of suppression caused.

Irrespective of why the precursor did not cause the probe detection threshold in the adaptation-of-suppression experiment to increase, the fact that it did not suggests that the observed overshoot was not associated with any appreciable increase in the signal response. In contrast, for enhancement, there is clear evidence that the precursor causes the signal response to increase (Viemeister and Bacon 1982; Byrne et al. 2011). However, this does not necessarily imply that enhancement is caused by adaptation of suppression. In fact, Wright et al. (1993) and Wright (1996) produced evidence against the adaptation-of-suppression account of enhancement. They measured suppression and enhancement in same set of participants and found a negative correlation between them; the adaptation-of-suppression hypothesis would predict a positive correlation. Furthermore, Viemeister and Bacon found, albeit with few participants, that the signal response was enhanced by a similar amount irrespective of whether the masker was actually present. Although Viemeister and Bacon speculated otherwise, it would generally be assumed that, when the masker was absent, the signal would not have been suppressed, and so, the observed enhancement could not have been caused by adaptation of suppression.

It is possible that enhancement is caused, not by adaptation of suppression, but, rather, by an increase in the responsiveness of the frequency channels within the spectral complement of the precursor. Thus, the mechanism of enhancement may be related to the mechanism underlying the Zwicker tone (Zwicker 1964; Lummis and Guttman 1972). The Zwicker tone is a faint tonal sensation following the presentation of a spectrally notched precursor similar to those used to produce enhancement, with a pitch in the range of the precursor notch. Wiegrebe et al. (1996) reported evidence suggesting that the Zwicker tone arises as a result of an increase in auditory responsiveness at frequencies corresponding to the Zwicker-tone pitch; at these frequencies, absolute hearing sensitivity was increased following the precursor presentation. The amount of increase in hearing sensitivity (up to 13 dB) was similar to the amount of increase in the signal response as a result of enhancement (Viemeister and Bacon 1982; Byrne et al. 2011). The Zwicker tone is an inconspicuous percept and can only be elicited at low or medium precursor levels. As a result, the precursor levels used by Wiegrebe et al. were much lower than those used in many enhancement experiments. Thibodeau (1991) showed that robust enhancement occurs up to very high precursor levels (91 dB SPL). It is currently not known whether the increase in absolute hearing sensitivity found by Wiegrebe et al. is limited to low and medium precursor levels like the Zwicker tone, or whether it persists at high precursor levels like enhancement. The properties of the Zwicker tone suggest that it arises centrally rather than peripherally. For instance, it is impossible to produce beating between the Zwicker tone and an external tone of a similar frequency (Krump 1993) and the Zwicker tone does not interact with spontaneous otoacoustic emissions (Wiegrebe et al. 1996). There is evidence suggesting that enhancement also arises centrally. Physiological studies have found enhancement in single-neuron responses in the inferior colliculus (Nelson and Young 2010) but not in the auditory nerve (Palmer et al. 1995). Psychophysical findings suggest that enhancement occurs, at least in part, beyond the point where the monaural pathways converge (Serman et al. 2008; Carcagno et al. 2012). More extensive characterization is needed to better understand the relationship between the two phenomena.

Enhancement causes an increase in the signal response, which, like the Zwicker tone, might be caused by an increase in auditory responsiveness within the spectral complement of the precursor, and which manifests as an increase in the signal’s forward-masking effectiveness. In contrast, in overshoot, any precursor-induced increase in auditory responsiveness would not be expected to change the signal’s forward-masking effectiveness. This is because of the short signal durations used in overshoot experiments. Any increase in auditory responsiveness would likely outlast the signal and equally affect the probe, leaving the probe detection threshold unchanged. The current study included a control experiment, which measured the probe detection threshold in the presence of the masker alone and the masker and precursor combined. Any precursor-induced increase in responsiveness to the probe should have resulted in a decrease in probe detection threshold. This however, was not observed.

Our results indicate that overshoot associated with a precursor and masker with energy only above the signal frequency is likely based on a different mechanism than enhancement. It is possible that enhancement only occurs when the precursor contains energy both below and above the signal frequency. This has been shown to be the case for the Zwicker tone, which is abolished when the lower and upper bands of the precursor are presented to different ears (Krump 1993, cited in Wiegrebe et al. 1996). Precursor and masker energy above the signal frequency accounts for the majority of off-frequency overshoot (Schmidt and Zwicker 1991). It is possible that off-frequency overshoot is caused by transient masking. Transient masking refers to the perceptual confusion between two transient events that occur close together in time (the short signal and masker onset in the case of overshoot; Bacon and Moore 1987). Alternatively, off-frequency overshoot may be caused by attentional diversion. Scharf et al. (2008) found evidence suggesting that the masker onset diverts attention away from the signal frequency towards the masker frequency and that the precursor mitigates this effect by allowing the listener to refocus attention back to the signal frequency. Both transient masking and attentional refocusing are higher-level effects, which, unlike enhancement, are unlikely to have correlates in subcortical processing.
